# Extensive parallelism in protein evolution

**DOI:** 10.1186/1745-6150-2-20

**Published:** 2007-08-16

**Authors:** Georgii A Bazykin, Fyodor A Kondrashov, Michael Brudno, Alexander Poliakov, Inna Dubchak, Alexey S Kondrashov

**Affiliations:** 1 Institute for Information Transmission Problems of the Russian Academy of Sciences (Kharkevich Institute), Bolshoi Karetny pereulok 19, Moscow, 127994, Russia; 2 Department of Ecology and Evolutionary Biology, Princeton University, Princeton, NJ 08544, USA; 3 Section on Ecology, Behavior and Evolution, University of California at San Diego, La Jolla, CA 92093, USA; 4 Department of Computer Science and Banting & Best Department of Medical Research, University of Toronto, Toronto, ON M5S 3J4, Canada; 5 Lawrence Berkeley National Laboratory, 1 Cyclotron Road, Berkeley, CA 94720, USA; 6 Department of Energy Joint Genome Institute, 2800 Mitchell Drive, Walnut Creek, CA 94598, USA; 7 Life Sciences Institute and Department of Ecology and Evolutionary Biology, University of Michigan, Ann Arbor, MI 48109-2216, USA

## Abstract

**Background:**

Independently evolving lineages mostly accumulate different changes, which leads to their gradual divergence. However, parallel accumulation of identical changes is also common, especially in traits with only a small number of possible states.

**Results:**

We characterize parallelism in evolution of coding sequences in three four-species sets of genomes of mammals, *Drosophila*, and yeasts. Each such set contains two independent evolutionary paths, which we call paths I and II. An amino acid replacement which occurred along path I also occurs along path II with the probability 50–80% of that expected under selective neutrality. Thus, the per site rate of parallel evolution of proteins is several times higher than their average rate of evolution, but still lower than the rate of evolution of neutral sequences. This deficit may be caused by changes in the fitness landscape, leading to a replacement being possible along path I but not along path II. However, constant, weak selection assumed by the nearly neutral model of evolution appears to be a more likely explanation. Then, the average coefficient of selection associated with an amino acid replacement, in the units of the effective population size, must exceed ~0.4, and the fraction of effectively neutral replacements must be below ~30%. At a majority of evolvable amino acid sites, only a relatively small number of different amino acids is permitted.

**Conclusion:**

High, but below-neutral, rates of parallel amino acid replacements suggest that a majority of amino acid replacements that occur in evolution are subject to weak, but non-trivial, selection, as predicted by Ohta's nearly-neutral theory.

**Reviewers:**

This article was reviewed by John McDonald (nominated by Laura Landweber), Sarah Teichmann and Subhajyoti De, and Chris Adami.

## Open peer review

Reviewed by John McDonald (nominated by Laura Landweber), Sarah Teichmann and Subhajyoti De, and Chris Adami. For the full reviews, please go to the Reviewers' comments section.

## Background

Although evolution is primarily divergent, parallel, convergent, and reversing changes in independently evolving lineages, collectively known as homoplasy, are not uncommon [1]. In particular, homoplasy should be pervasive when evolution is considered at the level of DNA or protein sequences, because there are only 4 or 20 possible states for each site. When evolving sequences become sufficiently dissimilar, homoplasious changes prevent their further divergence, leading to evolutionary saturation [2] and interfering with phylogenetic reconstructions [3]. Several instances of rapid parallel evolution of similar proteins, apparently driven by positive selection, have been observed (*e. g*., [4,5]). However, contribution of parallel changes to independent evolution of similar proteins has not been investigated quantitatively at the genomic scale.

Data on parallel amino acid replacements in proteins can shed light on several key aspects of their evolution. In particular, because the per site rate of nonsynonymous nucleotide substitutions *d*_N _is, on average, ~10 times smaller than the per site rate of synonymous substitutions *d*_S_, a vast majority of amino acids must be, most of the time, under substantial negative selection [2,6]. Still, neutral theory claims that most of amino acid replacements which occur in evolution are selectively neutral and, thus, must accumulate at the same rate as synonymous substitutions [6,7], as long as the latter are approximately assumed to be selectively neutral. The postulated relatively small proportion of rapidly evolving non-synonymous sites can be investigted using data on parallel evolution of proteins.

The minimal number of diverging sequences which makes it possible to study parallel evolution is four, because the phylogenetic tree must contain at least two nonoverlapping evolutionary paths, I and II. Here, we use sequences of three quadruplets of closely related genomes from mammals, fruit flies *Drosophila*, and yeasts *Saccharomyces*, and consider parallel evolution in these quadruplets at the level of all known proteins encoded by a genome. We define the rate of parallel evolution along path II as the rate with which allele replacements of a particular kind accumulated along this path, provided that the same replacements did occur, at the orthologous loci, along path I, and measure this rate for various kinds of amino acid replacements.

## Results

### Analysis of alignments

**Figure 1:**
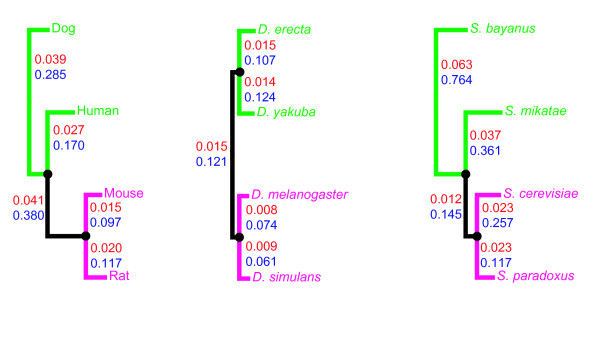
**Phylogenetic trees used in our analysis, drawn to scale**. For each edge, the average per site divergence at nonsynonymous (red) and synonymous (blue) sites is shown. Green lines show paths I, which are used to identify evolvable sites, and magenta lines show paths II, which are used to measure rates of evolution at these sites.

Phylogenetic trees used in our analysis are shown in Fig. 1. Evolutionary paths connecting human and dog, *D. yakuba *and *D. erecta*, and *S. bayanus *and *S. mikatae *are treated as "paths I", and mouse-rat, *D. melanogaster*-*D. simulans*, and *S. cerevisiae*-*S. paradoxus *paths, which are the shortest in the respective trees, are "paths II". If the two species connected by path I, and the two species connected by path II, display the same amino acid difference at the same site (say, amino acid A in one species from each pair, and amino acid B in the other species from each pair), parsimony implies that the same unordered amino acid replacement (A↔B) occurred along both paths I and II. In the case of *Drosophila *tree, with ((*D. yakuba*, *D. erecta*), (*D. melanogaster*, *D. simulans*)) topology (Fig. 1), we can be sure that replacements along both paths were parallel, *i. e*., occurred in the same direction (either A→B or B→A), although determining this direction is not possible. In the case of mammalian or yeast trees, replacements along path I and path II may also occur in the opposite directions, thus constituting a reversal; for example, a site with A in dog and rat and B in human and mouse can emerge due to A→B replacement in the lineage that led to the common ancestor of human, mouse, and rat, followed by B→A replacement in the rat lineage. However, the contribution of reversals should be relatively small, because the edge connecting nodes where dog and human lineages (or *S. bayanus *and *S. mikatae *lineages) branch off is rather short [8,9].

Informally, replacements along path I mark evolvable sites, and replacements, at these sites, along path II tell us how such evolvable sites evolve. Data on rates of parallel, and of coincident divergent, evolution of proteins are presented in Tables 1, 2, 3. In agreement with the previous estimates [10-12], the overall rate of nonsynonymous substitutions is ~10% of the rate of synonymous substitutions. In contrast, the average rate P of parallel nonsynonymous substitutions is much higher and constitutes ~50% (yeast), ~60% (mammals) or ~80% (*Drosophila*) of the rate of parallel synonymous substitutions. Assuming that the fitness landscape for an amino acid site did not change in the course of evolution represented by each tree, we can draw a number of conclusions from this figure.

**Table 1:** Divergence between mouse and rat at sites of divergence between human and dog

	Synonymous		Nonsynonymous			
		
	Overall^1^	Parallel^2^	Overall^3^	Coincident		
				
				Parallel^4^	Divergent^5^	
					
					Same site	Different site
Pairs of nucleotides						
AC	0.028	0.047	0.0028 (10.1%)	0.024 (122, 51.4%)	0.013 (280, 27.0%)	0.010 (306, 21.1%)
AG	0.089	0.109	0.0102 (11.5%)	0.069 (1570, 62.9%)	0.040 (448, 36.2%)	0.037 (923, 33.9%)
AT	0.021	0.024	0.0017 (8.4%)	0.028 (80, 117.4%)	0.008 (118, 32.6%)	0.006 (148, 23.5%)
CG	0.029	0.036	0.0033 (11.5%)	0.021 (156, 57.3%)	0.014 (302, 38.1%)	0.011 (332, 29.5%)
CT	0.076	0.090	0.0060 (7.9%)	0.051 (492, 56.5%)	0.027 (239, 30.3%)	0.020 (685, 22.6%)
GT	0.027	0.040	0.0019 (7.0%)	0.023 (69, 56.6%)	0.010 (175, 25.7%)	0.006 (168, 15.8%)
Average	0.045	0.058	0.0043 (9.6%)	0.036 (2489, 62.1%)	0.019 (1562, 32.2%)	0.015 (2562, 26.0%)
						
Genes^6^						
Low *d*_*N*_	0.043	0.059	0.0019 (4.3%)	0.028 (315, 47.1%)	0.012 (145, 21.1%)	0.009 (195, 14.8%)
Intermediate *d*_*N*_	0.046	0.058	0.0045 (9.9%)	0.036 (895, 61.7%)	0.017 (526, 29.6%)	0.013 (785, 21.8%)
High *d*_*N*_	0.047	0.057	0.0086 (18.5%)	0.038 (1279, 67.6%)	0.021 (891, 37.6%)	0.019 (1582, 32.7%)
						
Chemical distance between amino acids^7^						
1	0.045	0.058	0.0043 (9.6%)	0.038 (516, 65.0%)	0.012 (344, 20.1%)	0.016 (662, 27.3%)
1.5 or 2	0.045	0.058	0.0043 (9.6%)	0.033 (1537, 57.8%)	0.025 (834, 42.5%)	0.014 (1268, 24.7%)
2.5 or 3	0.045	0.058	0.0043 (9.6%)	0.031 (436, 53.7%)	0.023 (384, 40.4%)	0.018 (632, 31.0%)

^1^Mouse-rat divergence at all 4-fold synonymous nucleotide sites.

^2^Mouse-rat divergence only at those 4-fold synonymous sites where human and dog also underwent divergence in the same unordered pair of nucleotides.

^3^Mouse-rat divergence at all nondegenerate nonsynonymous sites. The magnitude of this divergence relative to overall synonymous mouse-rat divergence is presented in parentheses.

^4^Mouse-rat divergence at nondegenerate nonsynonymous sites where human and dog also underwent divergence in the same unordered pair of nucleotides and of amino acids. The number of sites of such mouse-rat divergence and the magnitude of this divergence relative to parallel synonymous mouse-rat divergence are presented in parentheses.

^5^Mouse-rat divergence at nondegenerate nonsynonymous nucleotide sites which belong to amino acid sites where human and dog also underwent nonsynonymous divergence, either at the same or at a different nucleotide site, but in a different unordered pair of nucleotides and of amino acids. The number of sites of such mouse-rat divergence and the magnitude of this divergence relative to parallel synonymous mouse-rat divergence are presented in parentheses.

^6^Genes were subdivided into three bins of equal sizes, according to their rates of nonsynonymous evolution along the path between mouse and rat.

^7^Rank of the Miyata distance between the human and dog amino acids among the distances between all pairs of amino acids that can arise due to a substitution at the same nucleotide site.

**Table 2:** Divergence between *D. melanogaster *and *D. simulans *at sites of divergence between *D. yakuba *and *D. erecta*.^1^

	Synonymous		Nonsynonymous			
		
	Overall^1^	Parallel^2^	Overall^3^	Coincident		
				
				Parallel^4^	Divergent^5^	
					
					Same site	Different site
Pairs of nucleotides						
AC	0.028	0.040	0.0025 (9.2%)	0.034 (110, 86.5%)	0.011 (99, 28.7%)	0.009 (119, 23.7%)
AG	0.055	0.070	0.0056 (10.3%)	0.053 (431, 75.3%)	0.023 (141, 32.7%)	0.020 (207, 28.8%)
AT	0.035	0.047	0.0024 (6.7%)	0.041 (119, 86.4%)	0.014 (93, 28.9%)	0.007 (74, 15.3%)
CG	0.022	0.031	0.0033 (15.0%)	0.036 (113, 114.9%)	0.011 (88, 35.0%)	0.010 (91, 31.2%)
CT	0.060	0.068	0.0037 (6.2%)	0.045 (152, 65.8%)	0.020 (103, 29.6%)	0.010 (98, 15.3%)
GT	0.027	0.036	0.0020 (7.6%)	0.032 (58, 88.1%)	0.010 (83, 27.9%)	0.010 (67, 27.2%)
Average	0.038	0.049	0.0033 (8.6%)	0.040 (983, 82.2%)	0.015 (607, 30.5%)	0.011 (656, 22.8%)
						
Genes^6^						
Low *d*_*N*_	0.035	0.048	0.0008 (2.3%)	0.026 (97, 53.8%)	0.006 (41, 13.6%)	0.004 (33, 7.9%)
Intermediate *d*_*N*_	0.038	0.048	0.0029 (7.4%)	0.037 (319, 77.1%)	0.012 (175, 25.4%)	0.009 (182, 18.5%)
High *d*_*N*_	0.041	0.051	0.0072 (17.5%)	0.046 (567, 90.9%)	0.019 (391, 37.5%)	0.015 (441, 29.1%)
						
Chemical distance between amino acids^7^						
1	0.038	0.049	0.0033 (8.6%)	0.048 (349, 98.2%)	0.008 (143, 16.7%)	0.010 (160, 19.8%)
1.5 or 2	0.038	0.049	0.0033 (8.6%)	0.037 (461, 75.7%)	0.016 (300, 32.0%)	0.011 (338, 21.8%)
2.5 or 3	0.038	0.049	0.0033 (8.6%)	0.032 (173, 65.4%)	0.019 (164, 39.3%)	0.015 (158, 30.6%)

^1^This Table is analogous to Table 1.

**Table 3:** Divergence between *S. cerevisiae *and *S. paradoxus *at sites of divergence between *S. mikatae *and *S. bayanus*.^1^

	Synonymous		Nonsynonymous			
		
	Overall^1^	Parallel^2^	Overall^3^	Coincident		
				
				Parallel^4^	Divergent^5^	
					
					Same site	Different site
Pairs of nucleotides						
AC	0.055	0.061	0.0037 (6.8%)	0.035 (132, 57.2%)	0.018 (217, 29.6%)	0.012 (223, 20.0%)
AG	0.198	0.218	0.0189 (9.5%)	0.110 (1941, 50.5%)	0.070 (459, 31.9%)	0.054 (851, 24.6%)
AT	0.044	0.064	0.0029 (6.6%)	0.038 (116, 59.2%)	0.015 (158, 24.0%)	0.006 (107, 9.2%)
CG	0.061	0.074	0.0053 (8.6%)	0.039 (117, 52.7%)	0.021 (214, 28.3%)	0.014 (129, 18.6%)
CT	0.169	0.208	0.0090 (5.3%)	0.095 (476, 45.7%)	0.044 (249, 21.1%)	0.025 (310, 11.9%)
GT	0.044	0.062	0.0025 (5.6%)	0.047 (76, 76.1%)	0.012 (143, 19.4%)	0.007 (64, 11.1%)
Average	0.095	0.115	0.0070 (7.4%)	0.061 (2858, 53.0%)	0.030 (1440, 26.2%)	0.020 (1684, 17.1%)
Genes^6^						
Low *d*_*N*_	0.085	0.110	0.0029 (3.4%)	0.049 (403, 45.0%)	0.015 (135, 13.7%)	0.011 (161, 10.1%)
Intermediate *d*_*N*_	0.101	0.117	0.0070 (6.9%)	0.062 (976, 52.6%)	0.027 (435, 23.0%)	0.018 (527, 15.2%)
High *d*_*N*_	0.103	0.117	0.0128 (12.5%)	0.064 (1479, 54.3%)	0.038 (870, 32.1%)	0.024 (996, 20.3%)
Chemical distance between amino acids^7^						
1	0.095	0.115	0.0070 (7.4%)	0.060 (969, 52.7%)	0.016 (308, 13.7%)	0.019 (434, 16.2%)
1.5 or 2	0.095	0.115	0.0070 (7.4%)	0.061 (1482, 53.0%)	0.032 (729, 27.9%)	0.018 (892, 15.4%)
2.5 or 3	0.095	0.115	0.0070 (7.4%)	0.053 (407, 46.7%)	0.040 (403, 34.6%)	0.032 (358, 28.0%)

^1^This Table is analogous to Table 1.

### Variability among individual sites

**Figure 2:**
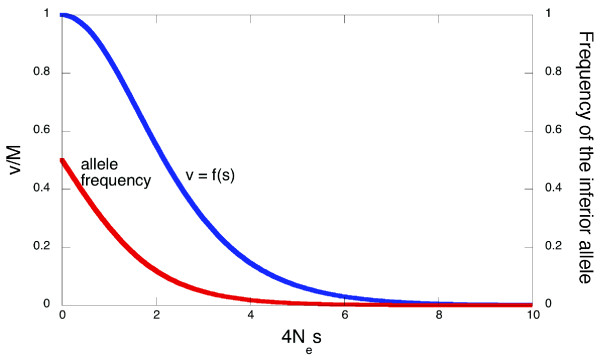
**Rate of evolution and frequency of the inferior allele as functions of 4N_e_s**. Rate of evolution v is in the units of rate of neutral evolution M [13].

Let us assume that only two alleles (amino acid variants) are possible at a locus (site). The selection coefficient associated with this pair of alleles is s = 1 - w_1_/w_2_, where w_1 _and w_2 _are constant fitnesses conferred by the two alleles (w_1 _< w_2_). Naturally, s can vary between 1 (lethality of the inferior allele) and 0 (selective neutrality). Under given parameters of mutation and the effective population size N_e_, the rate of evolution at the site v, *i. e*. the frequency of switches between fixations of the two alleles, is determined by s: v = f(s). When s is large enough (>>N_e _^-1^), the site is usually occupied by the best allele, and negative selection prevents fixation of the inferior allele. In contrast, when s < N_e _^-1^, either allele can be fixed at given moment. Assuming that the rates of forward and backward mutation are equal, v is the highest, and equals to the rate of neutral evolution M, with s = 0, and monotonously approaches 0 when s increases ([6,13]; Fig. 2). Consideration of only two alleles, involved in the observed parallel replacement, is sufficient because the ratio of frequencies of any two alleles under mutation-selection-drift equilibrium does not depend on fitnesses of any other possible alleles, as at such equilibrium the reciprocal fluxes of allele replacements are always equal [13].

Let us characterize all protein sites by their distributions of s, p(s), and of v, q(v). Then, q(v) = p(f^-1^(v)), where f^-1 ^is a function which relates s to the rate of evolution: s = f^-1^(v). The average rate of evolution at all sites is (all integrals are taken from 0 to 1)

C = ∫p(s)f(s)ds = ∫vq(v)dv

The distribution of s within sites where parallel evolution took place, *i. e*. the same replacement occurred along both short paths I and II, is given not by p(s) *per se*, but by p'(s) = p(s)f(s)/C, because the probability that a replacement occurred along path I is proportional to the rate of evolution at the site. Similarly, the distribution of v among such sites is q'(v) = vq(v)/C. Thus, the average rate of parallel evolution is

P = ∫p(s)f^2^(s)ds/C = ∫v^2^q(v)dv/C

**Figure 3:**
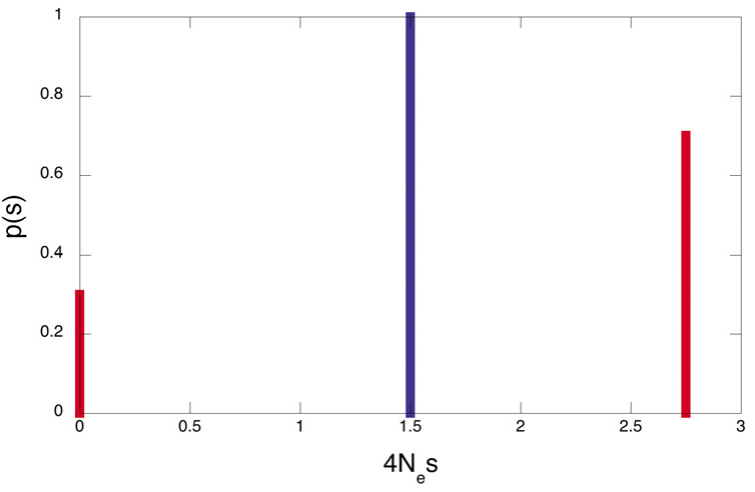
**Distributions of selection coefficients with extremal properties**. Distributions p(s) that correspond to the minimal average selection coefficient (blue line) and to the maximal fraction of selectively neutral sites (red lines), as long as the average rate of parallel evolution P constitutes 0.7 of the rate of neutral evolution. Vertical lines denote delta functions.

What does knowledge of P (say, 0.7; here and below P is given in the units of rate of neutral evolution M) tell us about p(s) or q(v)? The simplest option is that q'(v) and, thus, its "parent" distribution q(v), is concentrated at P = 0.7, and p(s) is concentrated at s_0 _= f^-1^(0.7) ~1.5/(4Ne) (Fig. 3). Of course, this cannot be the case, since different amino acid sites evolve at widely different rates [6]. Thus, the average value of s at sites of parallel evolution, S = ∫p'(s)sds, must be higher than f^-1^(P), because sites with above-average selection coefficients make smaller contributions to evolution than sites with below-average selection coefficients (Fig. 2). Assuming any particular shape of p(s) (*e. g*., that p(s) is a gamma-distribution with a particular shape parameter; [14]), we can calculate S which corresponds to the observed P.

Moreover, we can estimate the maximal fraction of effectively neutral sites (those evolving at essentially neutral rate 1) consistent with a given P. Indeed, the contribution of a site with some v = v_0 _to the reduction of P is (P-v_0_)v_0_. This contribution is maximal when d [(P-v)v]/dv = 0, *i. e*. when v_0 _= 0.5P. Thus, the fraction x of neutrally evolving sites is maximal, under a given P, when there are only two kinds of sites, those evolving with rates 1 and 0.5P. The average rate of evolution with such a q(v), [x+(1-x)P^2^/4]/[x+(1-x)P/2)], equals P when x = (P/(2-P))^2^. Thus, when P = 0.9, 0.8, 0.7, 0.6, and 0.5, the maximal possible fraction of effective neutral sites is 0.67, 0.44, 0.30, 0.18, and 0.11, respectively (Fig. 3). Of course, in reality this fraction must be lower, since p(s) and q(v) are not concentrated at just two points.

## Discussion

### Patterns in parallel evolution

Our data show that rate of parallel evolution of coding sequences is elevated: the probability of a change along path II is above-average at sites where the same nucleotide change also occurred along path I. This is the case even for synonymous substitutions: a synonymous substitution along path II is ~20% more probable when the same substitution also occurred along path I (Tables 1, 2, 3). As far as synonymous substitutions are concerned, between-sites heterogeneity of mutation rates and/or of the strength of selection can lead to the difference observed. Both mechanisms are feasible, because mutation rate varies between nucleotide sites [15] and because synonymous sites are not exactly neutral [16], and they are not mutually exclusive, but we did not attempt to determine their relative importance. The rate of parallel synonymous substitutions was used as a proxy for the rate of selectively neutral parallel nonsynonymous evolution.

The rate of parallel nonsynonymous substitutions P is elevated to a much larger extent than that of synonymous substitutions. Indeed, in the units of the rate of neutral evolution M, the average rate of all nonsynonymous substitutions is only ~0.1, but P is 0.5–0.8 (Tables 1, 2, 3). The sign of this difference is as expected: because rates of nonsynonymous substitutions are strongly heterogeneous across sites [6,17], a replacement observed along path I must be a good predictor of the probability of a replacement, at the same site, along path II. P is higher for rapidly evolving proteins or when a replacement involves two chemically similar amino acids. The rate of coincident divergent evolution, such that an amino acid replacement A↔C occurred along path II at an amino acid site where an amino acid replacement A↔B occurred along path I, is always much lower than the rate of parallel nonsynonymous evolution, but still higher than the average rate of all nonsynonymous substitutions (Tables 1, 2, 3).

Patterns in parallel evolution of proteins specifically reveal properties of only evolvable nonsynonymous sites. Indeed, the low overall rate of protein evolution implies that ~90% of all new nonsynonymous mutations are rejected by negative selection, but tells us little about sites where nonsynonymous substitutions do occur. In principle, if we assume that selection is constant, distributions p'(s) and q'(v), which characterize evolvable sites, can be derived from p(s), because f(s) is known theoretically (Fig. 2). However, this is currently impossible, because our data on p(s) are too crude. In fact, p'(s) depends only on the left tail of p(s), roughly corresponding to 4N_e_s < 10 (as sites under stronger selection do not evolve), and we know that this tail contains 10–20% of the distribution [18,19], but nothing definite about its shape. It is sometimes assumed that p(s) [14], as well as q(v) [20], are gamma-distributions, but these two assumptions cannot be simultaneously correct, and the issue remains obscure.

### Selection at evolvable sites

The best way to make sense of the data presented in Tables 1, 2, 3 is to consider possible reasons for a relatively small deviation of the average rate of parallel protein evolution P from the rate of neutral evolution M. Indeed, according to the neutral theory [6,7], most of accepted amino acid replacements are neutral, and in this case the two rates should coincide. The difference between P and M could be due to either variable or constant selection.

#### Variable fitness landscape

The only feasible scenario which could lead to P > 1 is positive selection caused by multiple changes in the fitness landscape. Only in this case can positive selection drive parallel replacements along both paths I and II. Indeed, two selection-driven parallel replacements can hardly be caused by a single change in the fitness landscape in the common ancestor of all the 4 species (Fig. 1), because a replacement must follow such a change with only a relatively short delay [6]. Therefore, at least two independent changes in the fitness landscape are necessary for parallelism – one in each lineage. Although rapidly fluctuating selection, resulting in *d*_N _> *d*_S _[21] and in parallel evolution ([4] and references therein) has been repeatedly observed, sites under such selection are rather rare, at least in mammalian genomes [22].

Thus, P < 1 is not surprising, and can be due to both variable and constant selection. If fitness landscapes are different along paths I and II, some replacements permitted along path I are forbidden along path II. In the extreme case of independent landscapes along the two paths, the rate of parallel evolution would not be elevated at all. In contrast to the case of P > 1, a single change of the fitness landscape, somewhere between paths I and II, is perfectly sufficient to explain P < 1.

A permitted replacement along path I can be either effectively neutral or driven by substantial positive selection. Two observations make the first option unlikely. Temporarily available opportunities for neutral evolution have been described by the covarion model [23], which assumes that selection at an amino acid site switches on and off as a result of amino acid substitutions elsewhere in the protein. Because the environment of an amino acid site is more stable within a slowly-evolving protein, covarion provides the least opportunity for path-specific neutrality and should lead to the smallest reduction of P in such proteins. However, we observed exactly the opposite: P was the lowest in proteins with low *d*_N _(Tables 1, 2, 3). Further, the evolutionary distance between paths I and II is much larger in the mammalian phylogeny than in the other two phylogenies (Fig. 1), which should lead to more on and off switches of selective constraint between the paths and, therefore, to stronger reduction in P in mammals. However, P is the lowest in yeasts, and not in mammals.

In contrast, the deepest reduction of P in slowly-evolving proteins is consistent with the hypothesis that positive selection plays a larger role in the evolution of slowly-evolving sites [24]. Still, it is hard to imagine that > 50% of all amino-acid replacements accepted by slowly-evolving proteins were adaptive, and this assumption is necessary to explain P < 0.5 in such proteins (Tables 1, 2, 3) through path-specific positive selection. Also, it seems implausible that changes of the fitness landscape favor radical replacements more frequently than conservative replacements, and the reduction in P is the strongest for substitutions which radically change the amino acid (Tables 1, 2, 3). Finally, the lowest rate of coincident divergent evolution, relative to P, in slowly-evolving proteins (Tables 1, 2, 3) implies that in such proteins the same pair of amino acids most often confers the two highest fitnesses along the whole phylogenetic tree, which appears to be inconsistent with frequent changes of the fitness landscape, unless such changes usually do not affect which two amino acids are the best.

#### Constant fitness landscape

The simpler assumption of constant selection seems to provide a more plausible explanation for the patterns observed. P < 1 is always expected if the two amino acids involved in the substitution are under constant selection (Fig. 2), as long as mutation is symmetric (see [16]). P = 0.7 is consistent with the average selection coefficient associated with an accepted amino acid replacement being 1.5/(4N_e_) = 0.375N_e _^-1 ^or more, and with the fraction of strictly neutral replacements being ~30% or less. In fact, selection on accepted replacements is probably even stronger, because we underestimated the rate of neutral evolution, which in mammals is ~10% higher, at nonCpG-prone sites, than the rate of synonymous evolution [16].

Thus, our data on parallel evolution of proteins suggest that a majority of amino acid replacements occur at sites which are not effectively neutral, but experience weak selection, in agreement with the nearly-neutral theory [25,26]. The highest P for replacements which involve the most chemically similar pairs of amino acids (Tables 1, 2, 3) is also consistent with this explanation, because such replacements must be under weaker selection than radical replacements.

If evolving lineages are at mutation-selection-drift equilibrium, the overall numbers of slightly deleterious and slightly beneficial replacements must be equal, although at a given moment a site is more often occupied by a (slightly) superior allele and, thus, is more often under negative selection. Still, at any moment, the fraction of amino acid sites occupied by slightly inferior amino acids must be well above ~10% of all evolvable protein sites (Fig. 2) and, thus, well above 1% of all ~10^7 ^protein sites of an organism. Selection with s ~10^-5 ^acting against > 10^5 ^slightly deleterious amino acids must cause a high genetic load [27].

### Sets of permitted amino acids at a site

Rates of coincident divergent evolution of proteins are ~3 times higher than their average rates of evolution, but still much lower than rates of parallel evolution (Tables 1, 2, 3). Indeed, rates of replacements, at an amino acid site, which involve different pairs of amino acids can be very different and must be analyzed separately [21]. Obviously, not every amino acid is permitted even at an evolvable amino acid site.

The average selection coefficient > 1.5/(4N_e_) associated with parallel replacements implies that the ratio of equilibrium frequencies of the two preferred amino acids at an amino acid site is ~4:1 (Fig. 2), assuming that the two amino acids involved in a parallel replacement usually confer the two highest fitnesses. Because for coincident divergent evolution P ~0.3 (Tables 1, 2, 3), the corresponding average selection coefficient should be > 2.5/(4N_e_) and the ratio of equilibrium frequencies of the preferred over unpreferred amino acid is ~20:1 (Fig. 2). Thus, rather roughly, a typical evolvable amino acid site should be occupied by the favored amino acid, the second best amino acid, and the other possible amino acids with probabilities ~75%, ~15%, and ~10%.

Of course, the sets of permitted amino acids vary greatly between sites. The rate of coincident divergence along path II is higher at amino acid sites where divergence along path I involves a pair of chemically dissimilar amino acids. Therefore, if two dissimilar amino acids are permitted at an amino acid site, other amino acids are more likely to be permitted as well. The number of permitted amino acids is known to vary widely across amino acid sites [28,29], and different sets of amino acids are permitted at different sites [30,31]. Data on parallel evolution, preferably along many independent paths, can be used to further investigate such sets.

## Methods

Orthologs of human, dog, mouse, and rat, as well as of four species of *Saccharomyces*, were identified by the bidirectional best protein BLAST [32] hit approach [33] using the Entrez retrieval system [34] of annotated protein coding genes from complete yeast and mammalian genomes, available at NCBI [35]. Alignments of amino acid sequences for each quadruplet were made using ClustalW [36] and reverse transcribed to obtain alignments of DNA sequences.

To align the whole genome assemblies of *D. melanogaster, D. simulans, D. yakuba *and *D. erecta*, we used whole genome multiple alignment algorithm implemented in the VISTA Genome Pipeline (Brudno *et al*. in prep.). This algorithm consists of two major modules – Pairwise Alignment of Sister Taxa and Progressive Multiple Alignment. First module uses a glocal (hybrid global/local) approach based on a reimplementation of the original Shuffle-LAGAN (S-LAGAN) chaining algorithm [37,38] combined with a post-processing stage called SuperMap. The S-LAGAN chaining takes as input a set of local alignments between the two sequences and returns the maximal scoring subset of these under certain gap criteria. In order to allow our alignments to incorporate duplications in both genomes, SuperMap algorithm takes two S-LAGAN outputs, for each sequence as the base. We then classified all local alignments as belonging to both chains, and consequently orthologous (best bidirectional hits), or being in only one chain, and hence a duplication. After the two pairs of sister taxa (*melanogaster/simulans *and *yakuba/erecta*) were aligned, we used a progressive generalization of the pairwise SuperMap algorithm to align the two alignments to each other, and get a 4-way alignment. Our algorithm is based on finding a maximum weighted matching in a graph, with the weights specified by the outgroup genomes, to order the individual alignment blocks in the likely order of the ancestors of *melanogaster/simulans *and *yakuba/erecta*. After that we align the resulting ancestrally-ordered alignments to each other using LAGAN [39]. To restrict our analysis to one-to-one orthologs, all cases in which an ORF in one species was aligned to more than one ORF in another species were excluded from the analysis. Since a substantial fraction of *Drosophila *coding regions had stretches of ambiguities and internal stop codons, we assumed that such stretches could have erroneous length due to sequencing errors. Therefore, if it allowed us to reduce the numbers of internal stop codons, we changed the lengths of stretches of ambiguities so to minimize the number of internal stop codons in the coding region.

*d*_S _and *d*_N _were estimated using pairwise nucleotide alignments taken from the four-species alignments for each pair of species using the codeml program of the PAML package [40]. To eliminate erroneous and nonorthologous gene alignments, those alignments in which pairwise *d*_S _and/or *d*_N _exceeded a pre-specified threshold between any pair of species were excluded from analysis. The thresholds were chosen manually to exclude the outlying alignments. The *d*_S _and *d*_N _values from PAML averaged over all the remaining genes were used as distances between species to produce the neighbor-joining phylogenetic trees in Fig. 1. Genes were split into three bins of equal size (low, intermediate and high *d*_N_) according to *d*_N _value between species of path II. The total numbers of quadruplets of orthologous genes analyzed were 11,105 for mammals, 3,735 for *Drosophila*, and 3,040 for yeasts. All the alignments and the Perl scripts used for analysis are available upon request.

To eliminate erroneous regions of alignments, which may originate from errors in genome assemblies or annotations, we only analyzed those codons that were flanked by gapless alignments of length ten codons or more from each side. To avoid the effect of hypermutability of CpG dinucleotide in mammals, we only included in the analysis of mammalian genomes nonCpG-prone sites, not preceded by C and not followed by G.

Synonymous divergence was estimated from alignments of fourfold degenerate sites, flanked from each side by a nucleotide conserved between all four species. We defined synonymous divergence between two species for an unordered pair of nucleotides (A, B) as the ratio of the number of sites at which one of species carries A and the other B, over the total number of sites at which they both carry A, both carry B, or one carries A and the other B.

Nonsynonymous divergence was estimated from nondegenerate nucleotide sites only, *i. e*. from sites at first or second nucleotide positions within an amino acid site at which each of the four nucleotides corresponds to a different encoded amino acid. In the analysis of divergence at the same nucleotide site, amino acid sites with divergence at more than one nucleotide site between any two of the four species were excluded from the analysis. In analysis of divergence at different nucleotide sites between paths I and II (*i. e*., species diverged along path I in first nucleotide site, and along path II at the second nucleotide site of the amino acid site, or *vice versa*) we required that only a single nucleotide be divergent between amino acid sites for each two species. Nonsynonymous divergence for a pair of nucleotides (A, B) was defined analogously to synonymous divergence.

Chemical distance between a pair of amino acids was taken as the corresponding term from the Miyata matrix [41]. The distance rank of a single-nucleotide nonsynonymous substitution between codons *c*_1 _and *c*_2 _took on values (1, 1.5, 2, 2.5, 3) and was calculated as the mean of the two values: 1) rank of the amino acid distance *d*(*c*_1_, *c*_2_) among all distances *d*(*c*_1_, *c*_n_), where *c*_n _are all one-point non-stop neighboring codons of *c*_1_; 2) rank of the amino acid distance *d*(*c*_1_, *c*_2_) among all distances *d*(*c*_m_, *c*_2_), where *c*_m _are all one-point non-stop neighboring codons of *c*_2_.

## Reviewers' comments

### Review 1 (John McDonald, University of Delaware, Dept. of Biological Sciences, Newark, DE, USA; nominated by Laura Landweber, Dept. of Ecology and Evolutionary Biology, Princeton University, Princeton, NJ, USA)

This manuscript compares three four-species sets of DNA sequences for coding regions. Each set of four species consists of two independent pairs. The main result is that there are fewer cases of parallel evolution for nonsynonymous sites than expected, based on the proportion of fourfold synonymous sites with parallel evolution. This is offered as evidence of mildly deleterious selection.

I think it is about time that homoplasy was treated as a source of evidence about evolutionary processes, not just an annoyance for systematists. However, I think the deficit of parallel evolution at nonsynonymous sites reported here may be an artifact, and the pattern may actually be explained by a "pure" neutral model, where the fitness of each mutation is either 0 or 1.

Consider the first line in table 1, which concerns sites that have A in mouse and C in rat, or vice versa. Of these AC sites in mouse-rat, 0.047 are also AC in human-dog. However, this 0.047 is only the fraction of human-dog sites "where human and dog also underwent divergence in the same unordered pair of nucleotides." Thus the denominator of the fraction yielding 0.047 is not the number of AC sites in mouse-rat, but the number of AC sites in mouse-rat, minus all sites with one or two non-AC bases in human-dog. Twofold neutral sites will all have A or C in human-dog, so the denominator will be larger (and thus the fraction of twofold neutral sites showing parallel evolution will be smaller).

***Author's response: ****There is a misunderstanding here. Table *1* describes the sites divergent between human and dog, as stated in the table heading. Contrary to what Dr. McDonald assumes, the first line in this table is concerned with sites that have A in dog and C in human, or vice versa. The phrase in the footnote that parallel sites are those "where human and dog also underwent divergence in the same unordered pair of nucleotides" is simply a restatement of this. 0.047 is the fraction of the sites with A-C synonymous divergence between mouse and rat, among all sites with A-C synonymous divergence between human and dog and either A or C in the common ancestor of mouse and rat. As described in Methods, the denominator of the fraction that yields 0.047 is the number of sites which have A and C, or C and A, in human and dog AND have A and A, A and C, C and A, or C and C in mouse and rat. We attempted to clarify this in the revised version of the manuscript*.

As a first approximation, we can assume that fourfold synonymous sites are fourfold neutral. For nonsynonymous sites, however, it seems likely that many sites with AC in mouse-rat will be only twofold neutral; the amino acids resulting from A or C at that position may have equal fitnesses, but the G or T amino acids may be strongly selected against. If that is the case, a smaller proportion of the twofold AC nonsynonymous neutral sites will show parallel evolution than fourfold AC synonymous sites, if the denominator excludes non-AC sites in human-dog.

Here's a numerical example to illustrate this. Consider a site with A in mouse and C in rat. The substitution probability is 0.1 to any other base between the mouse-rat ancestor and the human-dog ancestor (this is an unrooted tree), and 0.1 between the human-dog ancestor and either human or dog. If the site is twofold neutral, 0.19 of these sites will be AC in human-dog (2*0.1-0.1^2), ignoring multiple hits in a single lineage. So the proportion of twofold neutral AC sites in mouse-rat showing parallel evolution would be 0.19.

For the fourfold neutral case, 0.80 of the sites will have A or C in the human-dog ancestor. The proportion of sites that will be AC in human-dog is the proportion of sites with A or C in the human-dog ancestor, times the probability of a substitution to A or C in human or dog, times the probability that a substitution to G or T did not happen in the other lineage, or 0.80*(2*0.1-0.1^2)*(1-2*0.1) = 0.1216. Since the denominator is the proportion of sites that don't have G or T in either human or dog, it is the proportion of sites with A or C in the human-dog ancestor, times the probability that a substitution to G or T did not happen in either human or dog, or 0.80*(1-0.2)*(1-0.2) = 0.512. Thus the proportion of AC sites in mouse-rat that show parallel AC differences in human-dog, divided by the proportion of sites with AA, AC, or CC in human-dog, is 0.1216/0.512 = 0.2375. For the same mutation rates, 0.2375 of the fourfold neutral sites show parallel evolution, but only 0.19 of the twofold neutral sites do.

***Author's response: ****We do not see a problem here. In terms of Dr. McDonald's example, we did the following:*

*1) chose sites that experienced A-C divergence along path I (i. e., A in dog and C in human or vice versa)*,

*2) among such sites, chose those sites that had either A or C in the common ancestor for path II (assuming parsymony)*,

*3) for these sites, calculated the rate of A-C divergence along path II*.

*Assuming that only 1 substitution can occur alone path II at a site, we do not see how this analysis can be affected by the total number of permitted nucleotides at the site*.

I suspect that this artifact may not be large enough to explain all of the results in this manuscript, but the authors need to consider it. If the lower than expected proportion of parallel evolution for nonsynonymous sites holds up, this manuscript will be additional evidence that mildly deleterious evolution of nonsynonymous mutations is widespread.

One minor point – since the main result of this manuscript is that there is less parallelism in protein evolution than expected, the title "Extensive parallelism in protein evolution" is misleading; something like "Low parallelism in protein evolution as support for the nearly neutral model" would be better.

***Author's response: ****Indeed, our rate of parallel nonsynonymous evolution is ~30% below that of synonymous evolution, but it is also ~6 times higher than the average rate of nonsynonymous evolution. Thus, if one word is used to characterize this rate, "extensive" sounds appropriate*.

### Review 2 (Sarah Teichmann and Subhajyoti De, MRC Laboratory of Molecular Biology, Cambridge, UK)

The manuscript "Extensive parallelism in protein evolution" by Bazykin et al. provides an interesting insight into the parallel evolution at orthologus sites, and its effects on the observed rate of protein evolution. The work discusses how parallel accumulation of identical changes at equivalent sites contributes towards the effective rate of protein divergence, both under constant and variable fitness landscape. The authors conclude that the data favours a constant fitness landscape on the proteins they investigate.

The idea and results are an interesting molecular evolutionary study. However, we have some technical and conceptual comments, which need to be addressed to improve the manuscript.

#### Conceptual comments

1. The authors state that non-neutrality of synonymous sites and heterogeneity of mutation rates can influence the results on parallel evolution, but did not attempt to determine their relative importance. Can the authors provide a rough estimate of how much those factors can affect the final conclusions?

***Author's response: ****Because we use the rate of only parallel synonymous evolution as a proxy for mutation rate, heterogeneity of the rate of evolution across synonymous sites, due to heterogeneous mutation and/or selection, should not be a problem. However, uniform deviation of the rate of synonymous evolution from the mutation rate, caused by selection, cannot be taken into account in this way. Because we excluded hypermutable CpG sites (in mammals), it is likely that weak selection at synonymous sites could only decrease their rate of evolution, perhaps by ~10% (Kondrashov et al. J. Theor. Biol. 240, 616–626, 2006). If so, using the rate of synonymous evolution as a proxy for the rate of neutral evolution should cause a slight underestimation of the strength of negative selection at evolvable non-synonymous sites*.

2. The authors mentioned that coincident divergent evolution is always much lower than the rate of parallel nonsynonymous evolution. Presumably this is linked to the fact that evolvable sites will tend to switch to the most chemically similar amino acids. This is indicated by the Miyata distances in the tables, and the discussion section on permitted amino acids. However, the meaning of the percentages in the tables on the Miyata distances are not obvious to us, and are not explained in the table legend. To what extent is the ratio of parallel to coincident divergent sites explained by chemical similarity? What are the results using different chemical similarity measures?

***Author's response: ****We think that the explanation may be simpler. If, at a site, all the possible amino acids confer different fitnesses, substitutions that involve the best and the next best amino acid would be more common in evolution than any other, thus leading to the rate of parallel evolution being higher than the rate of coincident divergent evolution. The two "most preferred" amino acids do not necessarily represent the most similar pair at a site, although, on average, their Miyata distance tends to be short. As to the last point, very similar results where obtained using Grantham's similarity matrix (data not shown)*.

3. Along the same lines, are there examples of parallel evolution of sites that are very chemically different, and do they coincide with high dN/dS in a protein, indicating positive selection? Is there a general correlation of high dN/dS in a protein and chemical dissimilarity at coincident parallel sites? One could investigate this by considering individual proteins or groups of proteins rather than the entire data set.

***Author's response: ****This is indeed an interesting question. It appears from our data that proteins with LOWER dN/dS values on average allow for more radical parallel substitutions. However, there is insufficient data for this analysis to be included in the main text*.

4. The authors mention that the number of slightly deleterious and slightly beneficial replacements must be equal. When selection is non-zero at mutation-selection-drift equilibrium, it is not clear why this should be the case.

***Author's response: ****Assuming constant selection, the rates of reciprocal beneficial and deleterious replacements at mutation-selection-drift equilibrium must be the same, as long as we consider only 2 alleles – essentially, the definition of equilibrium. Less trivially, this fact holds even when we consider an arbitrary number of alleles (still under constant selection), because mutation-selection-drift equilibrium is a detailed equilibrium, with identical reciprocal fluxes of substitutions, and no cycling possible*.

5. Providing examples of parallel evolution of sites in a protein in the context of constant and variable fitness landscapes would be very helpful.

***Author's response: ****We would love to do this – but how can we be sure that fitness landscape was constant – or variable – for a particular protein?*

#### Technical comments

1. Although the biological aspects of equation (1) were clear, the mathematical parts could be elucidated more clearly: dv = d(f(s)) = f'(s)d(s), yet f'(s) is not present.

2. The authors talk about the conditions where P > 1 or P < 1 under variable and constant fitness landscape, and mention that the distribution q(v) is concentrated at P = 0.7. However it was unclear how the value is obtained. Is it simply derived from their results that P is between 50 and 80%? And what is the expected range of P?

3. The clarity of the manuscript needs to be improved to make it readily comprehensible to the broad audience, including aspects of English grammar. Subsections within in the 'Methods' section would improve the organisation of the manuscript.

4. Figure legends need to be more elaborate to improve clarity. Figure 3 can be omitted and instead described in a table or in the text. Instead, a figure of a protein with parallel mutations can be provided as a concrete biological example of the phenomenon.

5. How many parallel sites are there per protein on average in the three groups of four organisms? We cannot deduce this from any of the numbers in the manuscript.

***Author's response: ****We are thankful for these comments, some of which have been accomodated*.

The modified manuscript will definitely provide good insight into the parallel evolution leading to similar changes at identical sites and the evidence for weak negative selection on these sites in proteins. Once the above issues are carefully addressed, the improved manuscript is expected to meet the standard for publication in the Biology Direct.

### Review 3 (Chris Adami, Keck Graduate Institute, California Institute of Technology, Pasadena, USA)

In this paper, the authors study the parallel (and divergent) replacement of residues of proteins in different evolutionary paths, obtained by aligning sequences of three quartets of organism. The main observations are the following: the rate of parallel (non-synonymous) replacements is between 50% and 80% of the rate of parallel synonymous replacement, while the rate of divergent replacement (a residue replaced by another in one line, and replaced by a different one in the other line) is about between 25% and 30%. The authors conclude from this observation that most of the sites that undergo parallel evolution in these organisms (yeast, *Drosophila*, and mammal quartets) are under weak selection, as suggested by Ohta's "nearly neutral" theory.

There are clearly two parts to this paper: one is the assembly of data and their analysis to determine the rates of parallel evolution, the other is the interpretation of the data. I will limit my comments to the interpretation of the rates, and simply assume that the authors have assembled and analyzed the data with all due diligence.

The analysis of the rates of parallel evolution turns out to be rather tricky. The authors are mostly interested in determining the average selection coefficient at these sites. Clearly, if all sites were just evolving neutrally, then the rate of synonymous parallel evolution should be equal to the rate of nonsynonymous parallel evolution, i.e., P = 1. The question then is: how do you explain the reduced rate? Is the rate reduced because the two lineages are seeing different fitness landscapes (so that one residue is preferred in one lineage but selected against in the other), or is it because many sites actually have somewhat non-optimal residues to begin with, which are replaced by optimal ones in one, but not the other lineage?

If you assume that a variable landscape explains the suppression (i.e., P < 1), then you must assume that the change happened in the landscape of only one of the lineages, because if it happened in parallel for both, you'd see P > 1. If only one of the lineages sees a change in landscape, is the change neutral or driven by positive selection? The authors argue that it cannot be neutral because in this case slowly evolving proteins (for whom the landscape of "other mutations" changes only very slowly) should have a higher P, whereas a lower P is observed. Thus, the authors conclude that positive selection must be responsible for P < 1 if landscapes are changing.

***Author's response: ****So far, we agree with Dr. Adami*.

If landscapes are not changing, then the authors argue hat P < 1 implies slightly deleterious mutations at parallel sites. It is here where I have serious reservations. The reasoning is based on assuming that the average rate of parallel evolution reflects what is going on at single sites, that is, that the probability distribution of deleterious effects is narrowly distributed around the mean. But the authors themselves acknowledge that this is not the case (often a Gamma distribution is assumed), but then go on to say that the average effect of mutations at sites that undergo parallel evolution can nevertheless be calculated from knowing P. However, this reasoning also assumes that the distribution of P's across sites is narrowly centered around an average P, which again assumes a peaked distribution of deleterious effects. In other words, the conclusion that P < 1 implies a small mean effect of deleterious mutations is obtained by replacing distributions by their means, something that is exceedingly dangerous for distributions with an exponential tail.

***Author's response: ****Here, we cannot agree: we do not assume that the distribution of rate of evolution across sites, q(v), is narrow. We only claim that the average selection coefficient S is minimal when this distribution is narrow, so that any non-zero variance of q(v) leads to S > f-1(P) (see text). Moreover, we calculate the maximal fraction of selectively neutral sites that is consistent with a given P, under an arbitrary q(v)*.

While I cannot argue that the authors conclusion is necessarily wrong, it seems to me that in the absence of detailed knowledge about the distribution of deleterious effects, no conclusion can be drawn about how neutral or nearly neutral sites are that undergo parallel evolution. Indeed, it seems to me that positive selection is not out of the question, as a single change at one site can influence whether a change at another site is beneficial or deleterious (epistasis between mutations). Thus, rather than invoking environments being constant or changing for both lines, it is sufficient for one lineage to acquire a second mutation somewhere else in the protein in order for the change that is beneficial in the first lineage to be detrimental in the second. All else being equal, the ubiquity of epistatic interactions between mutations should favor an epistatic explanation of reduced parallelism.

It is also surprising that the authors do not discuss the evidence for parallel evolution accumulated in experimental evolution, where extensive parallelism is explained by overwhelmingly positive selection (Wichman et al, Science 285:422–424, 1999).

***Author's response: ****In the first paragraph of the paper, we cite several plausible instances of parallel evolution that is driven by positive selection. Generally, we agree with Dr. Adami that our arguments against variable fitness landscapes being the main reason for P < 1 are not water-tight. Still, weak, constant selection appears to provide the most plausible explanation for the data*.

## Authors' contributions

GAB designed the study and performed sequence analyses. FAK created mammalian and yeast sequence alignments. MB, AP and ID created *Drosophila *sequence alignments. ASK conceived and designed the study, performed mathematical analysis, and wrote the manuscript. All authors participated in writing, read and approved the final manuscript.
